# Comprehensive analysis of the influence of loess structure on strength on the Loess Plateau, China

**DOI:** 10.1371/journal.pone.0333204

**Published:** 2025-09-29

**Authors:** Hui Cheng, Hong Li, Shaotong Jiao, Youshan He, Junfeng Deng, Huan Wei, Yixuan Song, Rui Xu

**Affiliations:** 1 PowerChina Northwest Engineering Co. Ltd., Xi’an, China; 2 Chengdu University of Technology, Chengdu, China; 3 School of Geology, Engineering and Geomatics, Chang’an University, Xi’an, China; Institute of Disaster Prevention, CHINA

## Abstract

This study examines the influence of loess structure on strength by selecting three representative regions of the Loess Plateau in northern Shaanxi as the research area. Malan loess serves as the test subject, and in-situ direct shear tests, particle flow modeling, and theoretical analysis are employed to investigate the impact of loess structure on the damage process of soil shear deformation and its internal mechanisms. The numerical simulation examines how the alteration of clay particle content, within a range of 1%−10%, affects the strength variation of the soil, as indicated by the in-situ test. The results indicate significant geographical heterogeneity in loess strength indices, with the structural properties of loess being a primary factor contributing to this variability. The primary trend indicates an increase in clay content of loess, a decrease in grit content, a relative increase in cohesion, and a relative decrease in the internal friction angle. Furthermore, the variation in cohesion is more pronounced than the internal friction angle. This article examines the relationship between loess strength and its structural properties, enhancing the concept of strength analysis. The findings will offer a theoretical foundation for investigating regional variations and the regionalization of engineering design in the loess area of northern Shaanxi.

## Introduction

Loess is widely distributed throughout much of China, Russia, Europe, North America, and New Zealand [1–3]. China exhibits the most extensive distribution, most significant thickness, and most pronounced geomorphology of loess globally, comprising 6 percent of the nation’s land area, with the Loess Plateau region being the most representative [4]. Loess in the Loess Plateau region is primarily made up of clay, powder, and grit, and the distinctive engineering geological characteristics are intimately associated with the loess structure. The unique generation environment has imparted distinct structural properties to the loess on the Loess Plateau, specifically regarding the size, shape, arrangement, and association of soil particles, as well as the geometry of the particles and the interstitial pores. In its natural state, loess has relatively high strength and engineering properties.

Unlike other soils, loess has developed joints and fissures; the macroscopic structure is similar to the karst characteristics, and the microscopic structure is loose and porous. The difference between dry and wet strength is substantial; changes in the aquatic environment can easily lead to a loess tragedy. It has brought many challenges to infrastructure construction, such as uneven settlement of roadbeds, eolian landslides, pit slides, tilting, and even the collapse of buildings and other disasters [5–7]. To reveal the nature of the physical-mechanical behavior of compacted loess, it is necessary to be clear about its internal microstructural changes. Therefore, it is crucial to study the influence of loess structure on its strength.

Loess in the Loess Plateau region consists mainly of clay, grit, and powder, which exhibit favorable engineering qualities and strength properties in their natural condition [8–10]. However, loess is a soil in which the interaction of soil particles is highly susceptible to external factors, and soil strength is strongly correlated with structural characteristics such as particle composition. Loess structural damage, for example, causes soil damage and wetting phenomena [11–12].

Although scholars at home and abroad have also carried out many studies on the correlation between soil structure and soil strength [13–14], the studies are mainly on the influence of external factors such as water content and compactness on the mechanical index of soil. Some of these studies have also focused on the impact of internal factors, such as soil particle composition, and attempted to use conventional indoor mechanical tests, numerical simulation, and theoretical analyses to study the mechanical characteristics of the soil structure. However, the specimens in these investigations were prepared using artificially remolded or modified soils. Although the controllability of the variables and the relevance of the experimental data can be considerably enhanced, it is still challenging to portray the structure’s depositional history and the soil structure’s actual molding by environmental conditions [15–16].

Conversely, the transfer of soil samples from the field to the laboratory may disturb the soil, altering its structure and properties, compromising the test results’ accuracy [17–20]. Environmental factors may influence soil characteristics, including temperature, humidity, and pressure [21]. While laboratory conditions might not accurately replicate these factors, in-situ tests can be conducted more closely to realistic environmental conditions. Thus, under particular environmental circumstances, in-situ mechanical tests remain a straightforward and efficient way to investigate the stress damage process of loess structures [22–25], and serve as a crucial foundation for theoretical and indoor experimental investigations.

In view of this, this paper selects three typical test sites on the Loess Plateau in northern Shaanxi, and uses an independently designed large-scale in-situ straight shear test system to study the changing law of loess strength characteristics; and then uses the particle flow discrete element to simulate the loess shear deformation and damage process to illustrate the influence of structural characteristics of loess, such as the content of clayey grains, on the loess mechanical properties of the mechanism. The study results show that the different mechanical properties exhibited by other particles, such as clay, powder, and sand, in the process of force deformation and damage of the soil directly affect the strength index of the soil.

## Materials and methods

### Overview of the study area

The Loess Plateau in northern Shaanxi is a pivotal node and vital hub of the “Belt and Road” initiative, as well as the most significant strategic reserve base for coal, oil, gas, and other energy resources in China. The Loess Plateau is mainly mountainous, with broken topography, longitudinal gullies, and loess strata. The northern Loess Plateau region of Shaanxi, with Yulin, Yan’an, and Qingyang as its core, is one of the regions in China with the most prominent contradiction of human-land coupling, the most fragile ecological environment, and the most severe geologic hazards [14–15]. Given this, this paper sets the experimental research area in unoccupied field slopes adjacent to oil field station sites of Qingyang, Yan’an, and Yulin, respectively, and selects the original Q3 Malan loess for the test site. The test soil layer can be obtained at the experimental site by excavating an exploratory pit or flat on the slope platform of the excavated slopes next to the oil field station. This study was conducted in an uninhabited area. No human participants were involved, and therefore, no approval from an institutional review board was required. The soil samples are mostly light grayish-yellow in case the site is not affected by rainfall, and the mean values of the physical property indexes are shown in Table 1.

**Table 1:** Indicators of physical properties of soil samples.

Location	Specific gravity (Gs)	Natural density *(ρ/g·cm*^*-3*^)	Natural moisture content *(w/%)*	Natural pore ratio *(e)*	Liquid limit *(w*_*L*_*/%)*	Plastic limit *(w*_*P*_*/%)*	Saturation *(S*_*R*_*/%)*
Qingyang	2.68	1.49	12.9	1.043	24.4	16.1	33.38
Yan’an	2.69	1.51	11.3	0.985	23.9	15.8	30.54
Yulin	2.70	1.52	10.9	0.968	24.1	15.7	32.97

### Test system design

**Fig 1:**
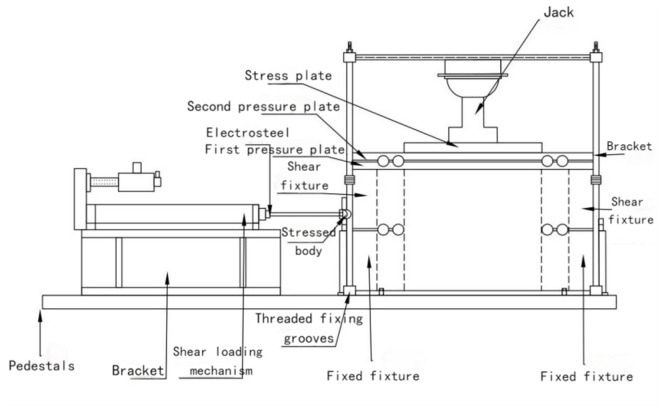
Direct Shear Experimental System.

**Fig 2:**
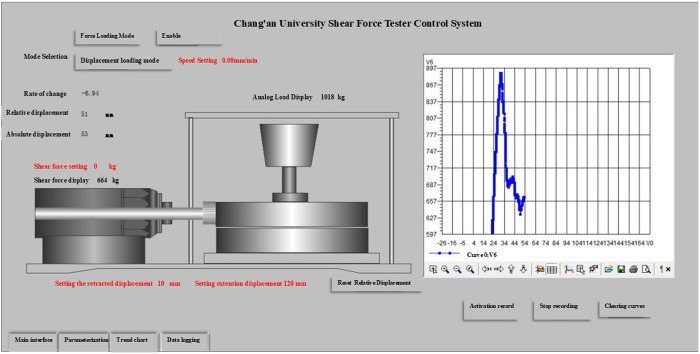
Human-computer interface.

As shown in Fig 1, the project team designed and manufactured the test apparatus and program specifically to meet the unique environmental conditions in northern Shaanxi Province. The shear experiment system consists of a base, a support frame, a fixture, a loading and unloading mechanism, an electrical control cabinet, a computer, and software. The data acquisition system comprises sensors, controllers, a CPU, drives, motors, other hardware, and custom software. The software displays, saves, records, and prints data; Fig 2 depicts the port interface for human-computer interaction. The power system includes an electric cylinder, a load, and a displacement measuring device.

The test equipment delivers various everyday stresses to the soil in the natural stratum via the shear system while monitoring the shear displacement and resistance relationship curves, providing immediate access to the peak and residual strength data. In contrast to the conventional indoor testing approach, in-situ testing circumvents issues related to structural deterioration, alterations in water content, and stress release resulting from sample disturbances. It is particularly appropriate for coarse-grained soils, fissured clays, and other specialized soils that are challenging to sample. Its primary advantage is preserving the soil mass’s natural structure and stress state through in-situ testing, accurately reflecting the actual stress response of the soil layer at the engineering site. Simultaneously, owing to the “boundary effect,” in situ large-scale straight shear experiments reveal that loess particles are more predisposed to establish a complete force chain structure than indoor tiny straight shear tests, resulting in measured data that more accurately reflects the actual state of loess.

The test system is highly modular; each component can be disassembled, and the entire mechanical device is a combined structure with mutual positioning planes, which are easy to assemble and disassemble many times. A bolt connection can complete the assembly and disassembly work with standard tools. The quality of the base plate is 70 kg, and the quality of the rest of the single parts is less than 30 kg. The size is 1300 × 720 × 750 mm, the whole is small, and one or two people can complete the transportation and assembly. The experimental system’s design focuses on reducing friction during shearing, which is accomplished by using fixed welding between each fixture and the platen, as well as a custom-designed sliding ball and slide structure. In addition, the system includes a retractable stand that can be adjusted within a specific range. This design enables the experimental setup to be adapted to different geological environments, including sloping platforms and flat caves. Due to the small space required, the system is suitable for flat cave environments and can also be operated on narrow sloping platforms, providing excellent adaptability and flexibility.

**Fig 3:**
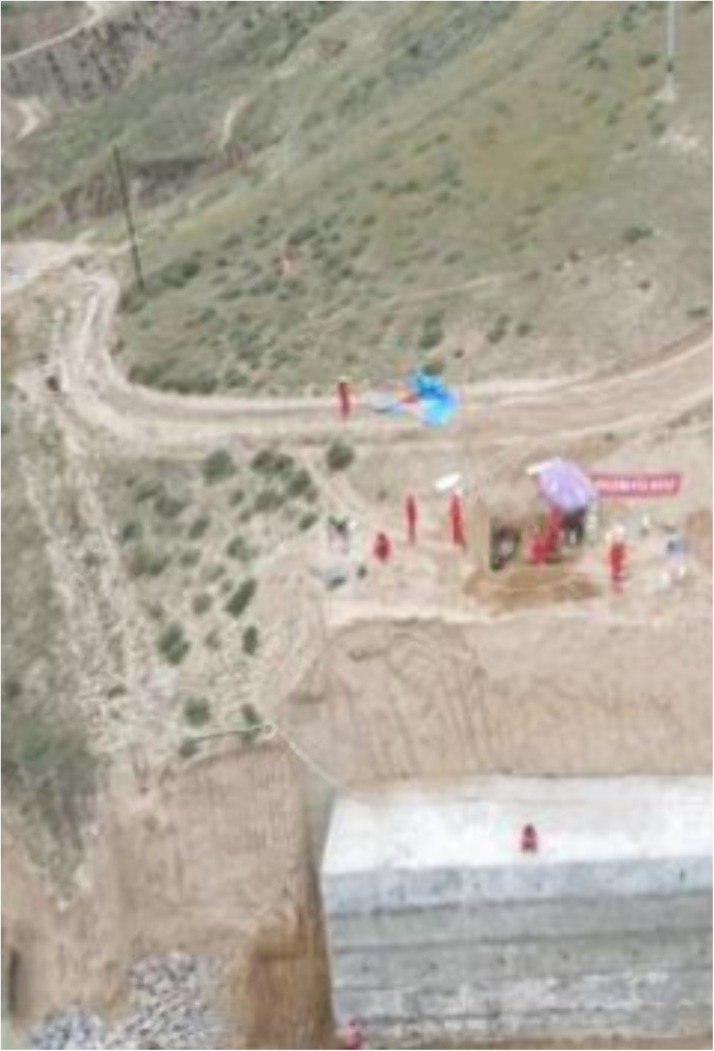
Trial pits.

**Fig 4:**
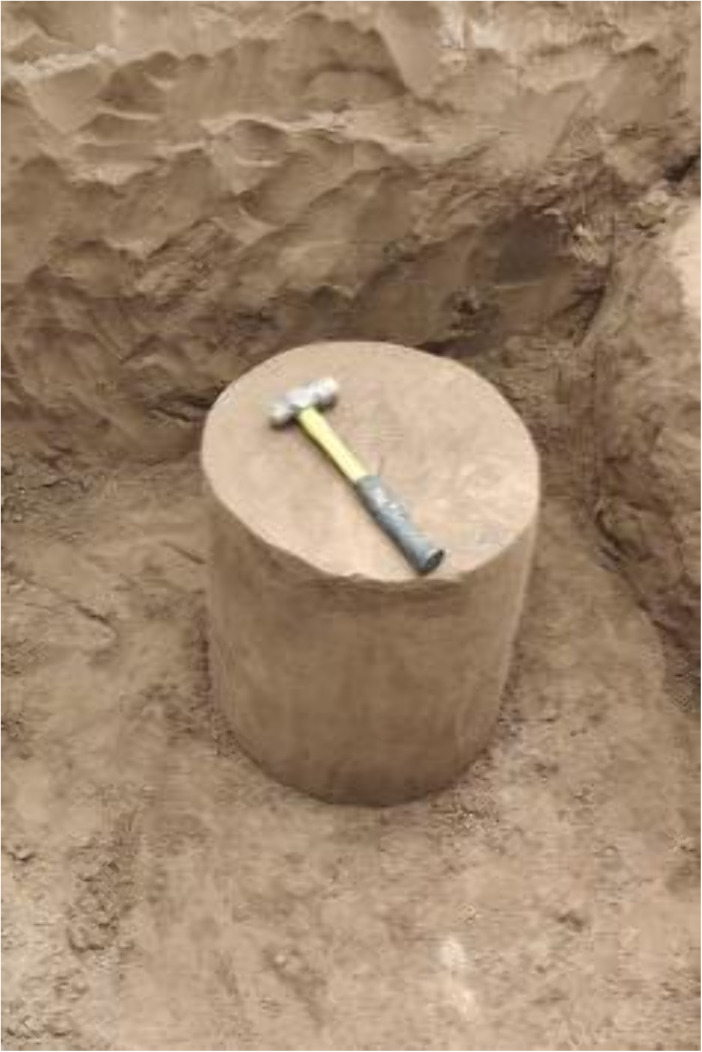
Specimen making.

**Fig 5:**
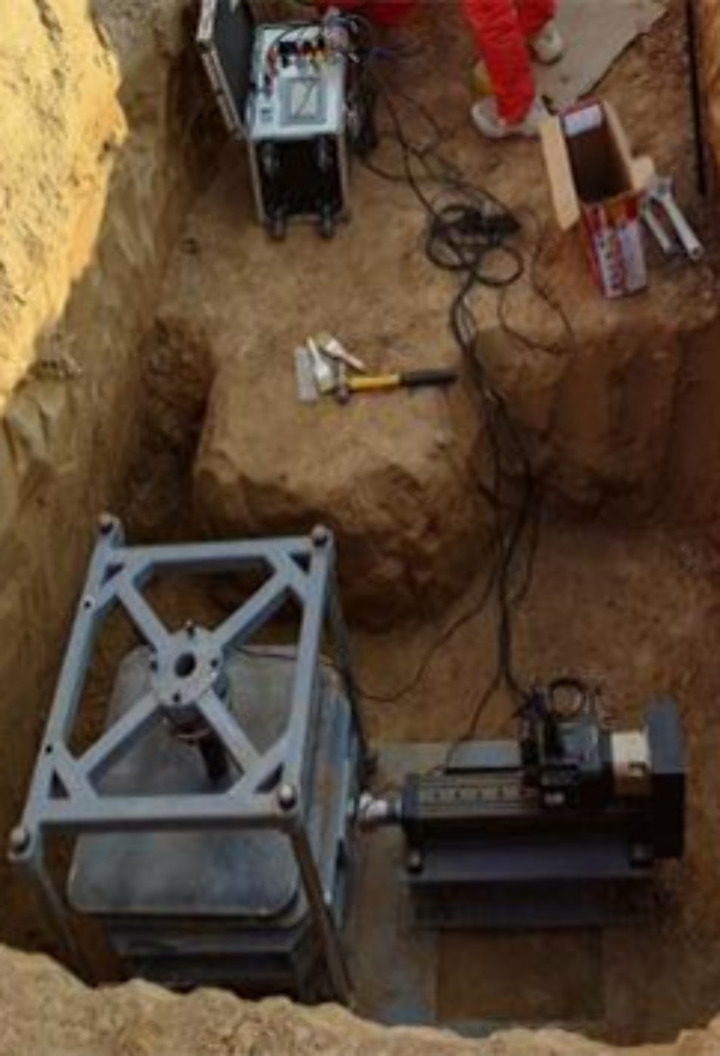
Equipment installation.

**Fig 6:**
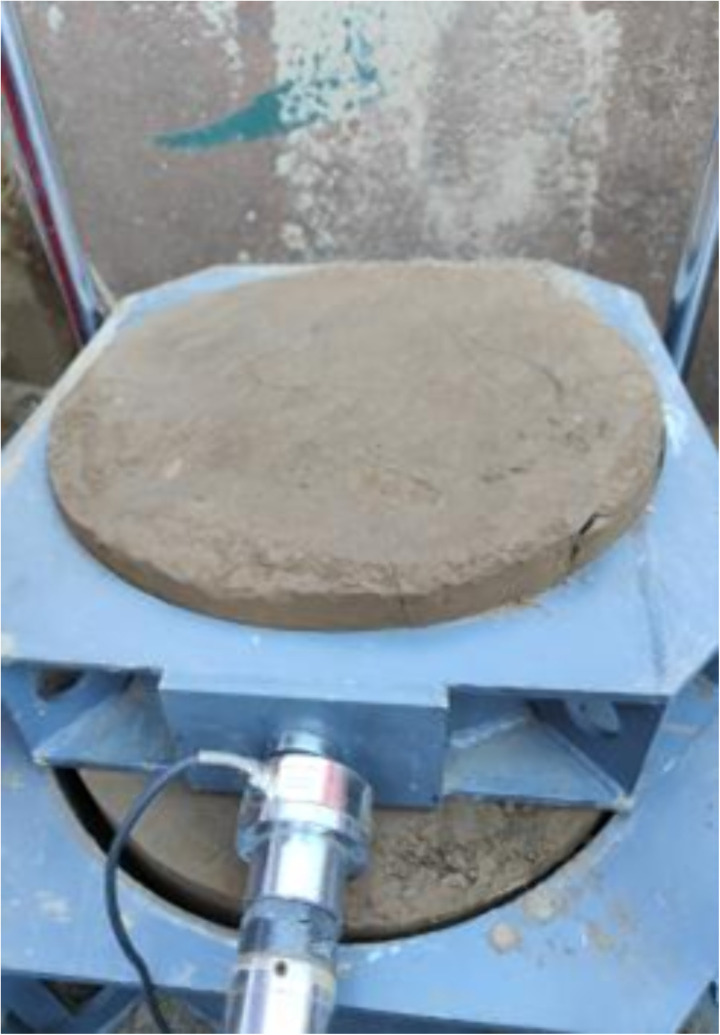
Shear completion.

**Fig 7:**
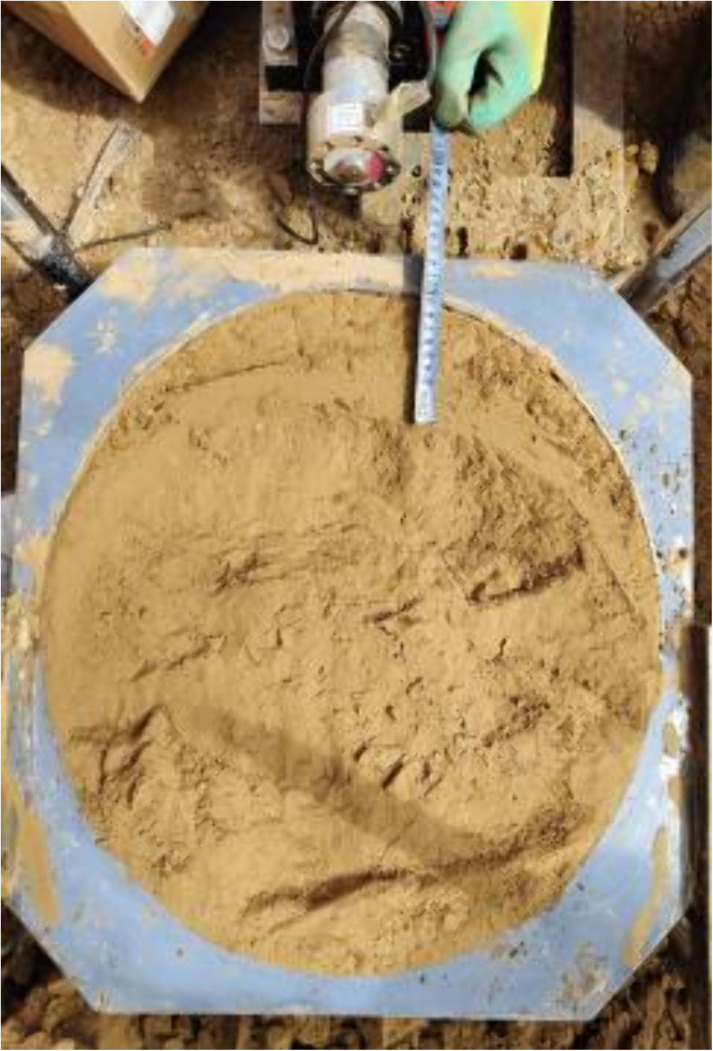
Recording of results.

The specific steps of the straight shear test are shown in Figs 3–7: As shown in Fig 3, after selecting the experimental site, a probe pit or flat cave must be excavated to make soil samples. (After the experiment, the pit or refuge on the slope will need to be backfilled to minimize ecological impacts.)

The precise procedures of the straight shear test are illustrated in Figs 3–7: As described in Fig 3, based on the spatial arrangement of structural surfaces inside the geotechnical mass and the orientation of the intended shear, a compass and tape measure are employed to measure and position the test pits in the field, as well as to determine the alignment of specimen arrangement. Test pit excavation may only proceed following the preliminary identification of the target stratum by exploration, ensuring the representativeness of the test results.

The in-situ soil samples were collected following the identification of the pit position for sampling and the establishment of the support framework, informed by the properties of the ground structure, excavation dimensions, and test loads. Soil samples must be obtained from in-situ soils beneath the bottom 0.3 meters. The in-situ soil samples possess approximate cylindrical dimensions, with a diameter of d = 50 cm and a h = 55 cm. The polygonal initial sample is cut, followed by the manual formation of the standard cylinder by a meticulous fine-cutting process.

After the soil sample was successfully chipped, the device was installed as shown in Fig 5. Subsequently, the horizontal shear speed and vertical load parameters were set on the electrical control cabinet, and the shear test was carried out to record the data. The *Code for investigation of geotechnical engineering (GB 50021−2001) s*tipulates a shear rate of 0.08 mm/min. Monitor the shear process and terminate the experiment, preserving the data, upon reaching the peak value or at the failure of the soil sample due to shear. The recording process is shown in Fig 2. As in Fig 6, the experiment was stopped when the peak value was reached or the soil sample was damaged by shear, as in Fig 6. The shear image of the field test soil sample was retained to save the data.

### Composition of loess particles

**Fig 8:**
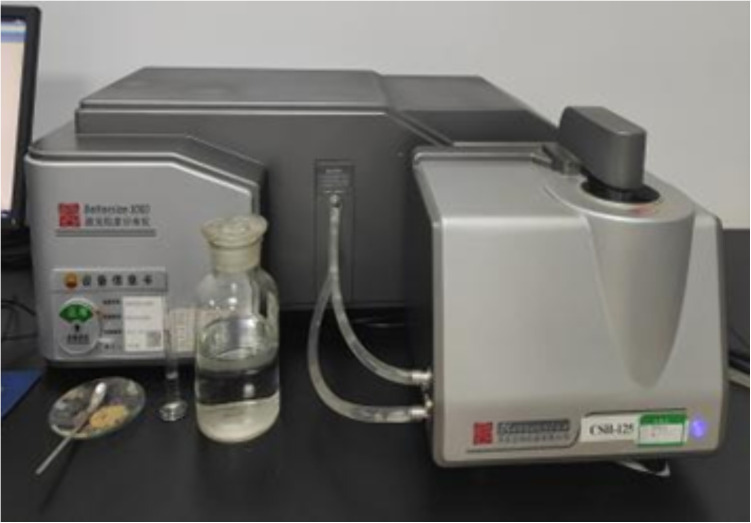
Laser particle size distributor experiment test equipment.

**Fig 9:**
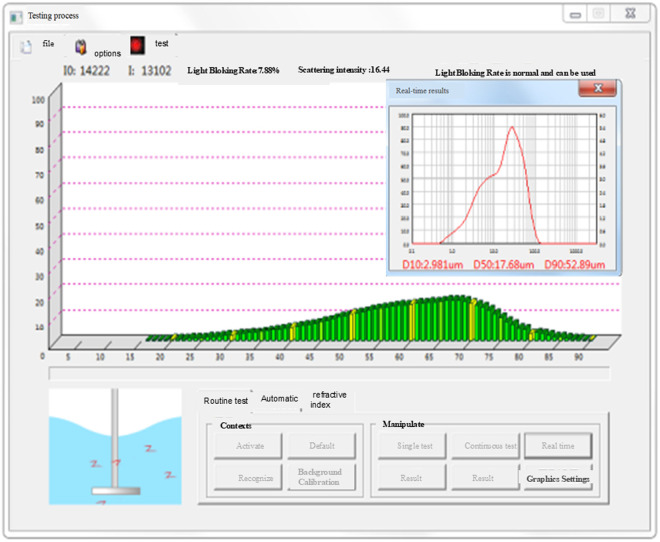
Laser particle size distributor experiment test procedure.

**Fig 10:**
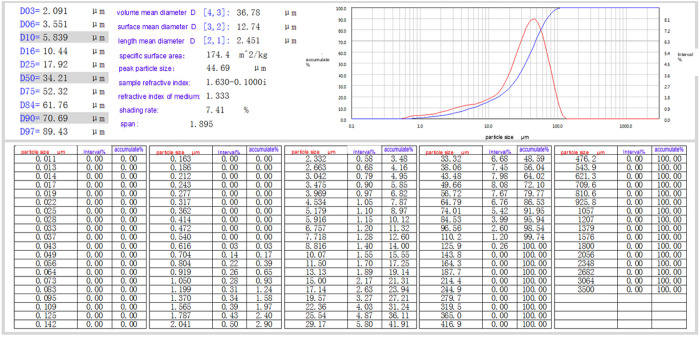
Laser particle size distributor experiment results.

The particle analysis test of soil samples was also carried out on this occasion to analyze prominently the law of influence of loess structural features, such as clay particles, on the strength characteristics of loess. During the experiment, the loess samples were first pre-treated appropriately to ensure the accuracy of the test. The samples were then analyzed using a laser particle size distribution instrument, which emitted a laser beam. When the laser beam came into contact with the particles, it was scattered, and the instrument used the angle and intensity of the scattered light to calculate the particle size. This advanced testing method allows for a detailed analysis of the clay content of loess samples as well as the distribution of other particle sizes. This particle analysis test was performed with a Bettersize 3000 Laser Particle Size Distribution Instrument, as illustrated in Figs 8–10.

### Numerical model

PFC^3D^simulates granular materials via discretization, treating each granule as an individual particle that interacts through contact forces, rendering this simulation method appropriate for investigations concerning the structural composition of loess.

**Fig 11:**
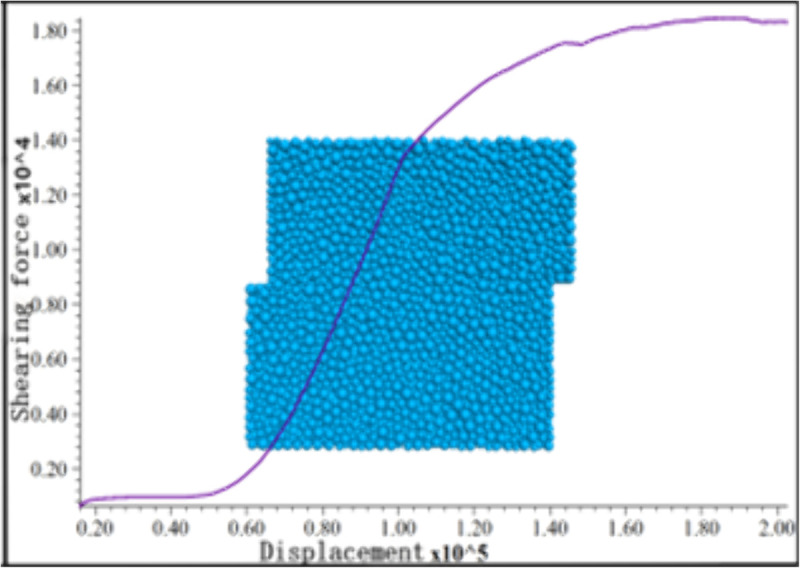
Discrete element simulation.

To analyze the relationship between soil strength and soil structure from the fine-level analysis, this paper is based on the basic theory of discrete elements [15–18], and the shear test model is established through the PFC3D three-dimensional particle flow program [19] to simulate the in situ straight shear test. The numerical simulation process is shown in Fig 11. The boundary conditions and parameters were set with reference to in situ experimental data, theoretical analyses, or previous studies [16] to ensure the reliability of the simulation results. Furthermore, other numerical simulation models with varying viscous particle contents are established in this paper. Through the built-in FISH language of PFC^3D^ for particle deposition, particle repulsion, adjusting the particle velocity to stabilize the initial imbalance force between the particles and avoid the overlapping of the particles, and finally performing the shear test after the specimen is solidified, the particle flow model for each condition is used to generate the specimen particles according to the preset particle gradation.

Soil data were obtained from practical studies and included in the numerical simulation model, as illustrated in Table 2.

**Table 2:** Numerical simulation setup parameters.

Characteristics of the Model	Property parameter	Value
Bonding models	Emod(Mpa)	8
Contact force parameters	Stiffness ratio	1
	Friction coefficient	0.28
	Damping factor	0.7
Particle size distribution	PPorosity	0.55
	Density $\left(kg/m^{\text{3}}\right)$	2710

## Results

### Shear damage process

**Fig 12:**
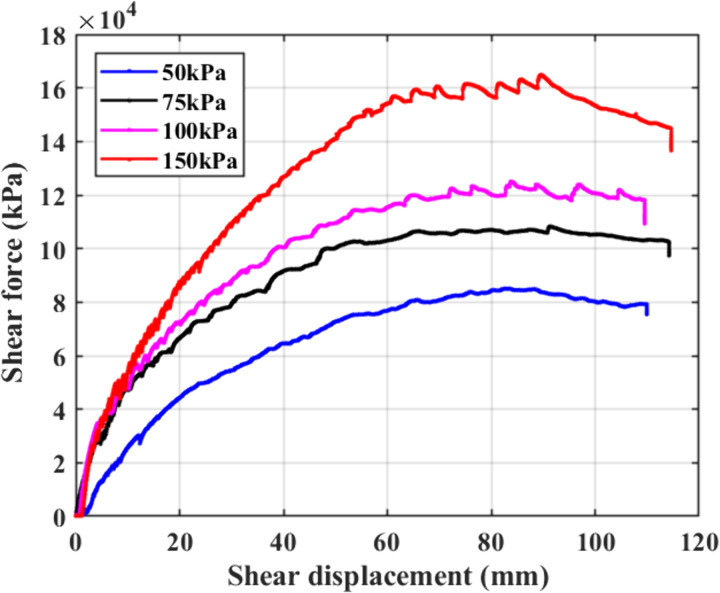
Shear displacement curve.

Fig 12 shows the relationship between the shear force and the displacement of a typical set of in-situ straight shear test statistics.

According to Fig 12, the specimens exhibit strain-hardening properties under various normal pressures, and it is evident that the transverse shear force increases more at the start of the test. The curve has a “yielding” general trend and “stepped” stages under various normal pressures. The soil will first reach the yielding state under lower loads, as indicated by the curve’s earlier entry into a smooth trend at lower normal pressures (such as 50 kPa and 75 kPa). The deformation of the surrounding soil is observed during the experimental process. As shear deformation increases rapidly, the shear force rises at a reduced rate and stops increasing upon reaching its maximum value. Without a peak, a soil sample deformation of 1/10 signifies the threshold at which the soil has sustained damage; at this juncture, the associated shear force can be regarded as the maximum shear force.

**Fig 13:**
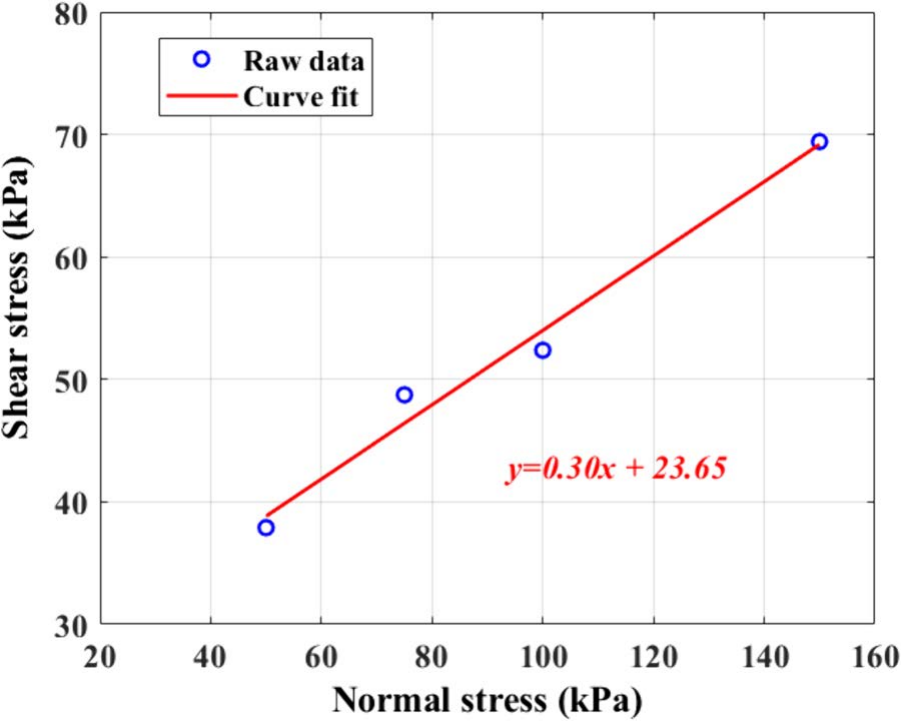
Intensity curve.

The shear force obtained from the test is converted to shear stress, which can be obtained as the soil’s shear strength index under various normal pressures, and the relationship curve between shear stress and normal stress is plotted to obtain the strength curve, as shown in Fig 13, and the strength parameters, such as cohesion and internal friction angle, are determined.

### Strength index of loess

**Fig 14:**
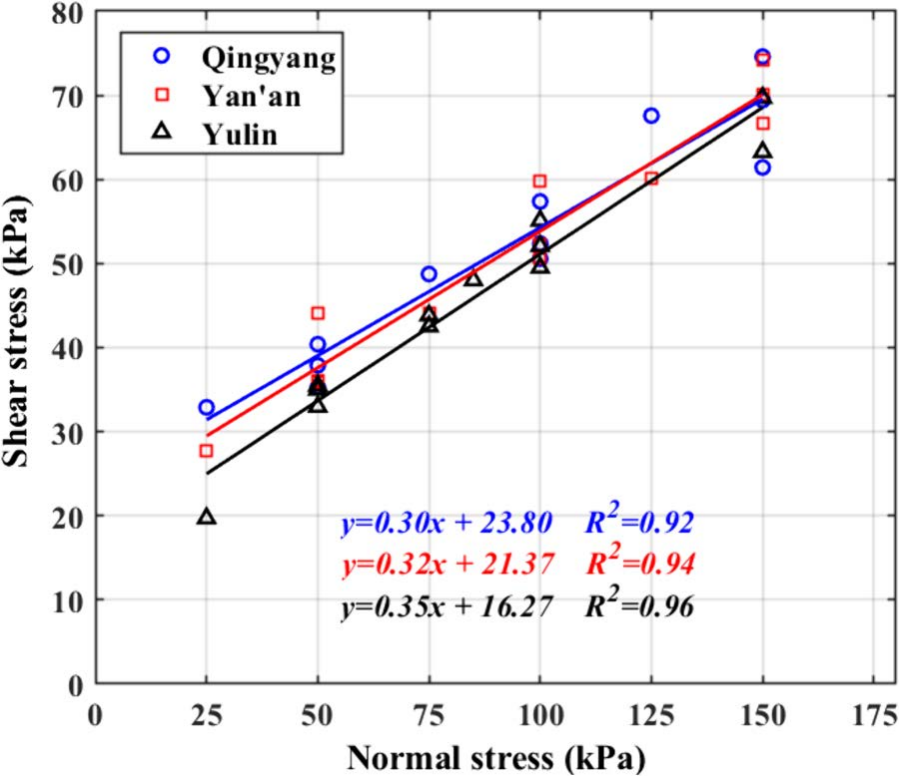
Intensity profile (total).

The group fitted the stress and shear stress distribution laws obtained from 36 large-scale in situ straight shear experiments conducted at three experimental sites, as shown in Fig 14.

By organizing the fitted curves, the shear strength of the soil in the three regions was obtained, as shown in Table 3.

**Table 3:** Results of shear resistance experiments.

Location	Cohesion*/kPa*	Internal friction angle *φ/°*
Qingyang	23.80	16.96
Yan’an	21.37	17.99
Yulin	16.27	19.21

### Composition of loess particles

**Fig 15:**
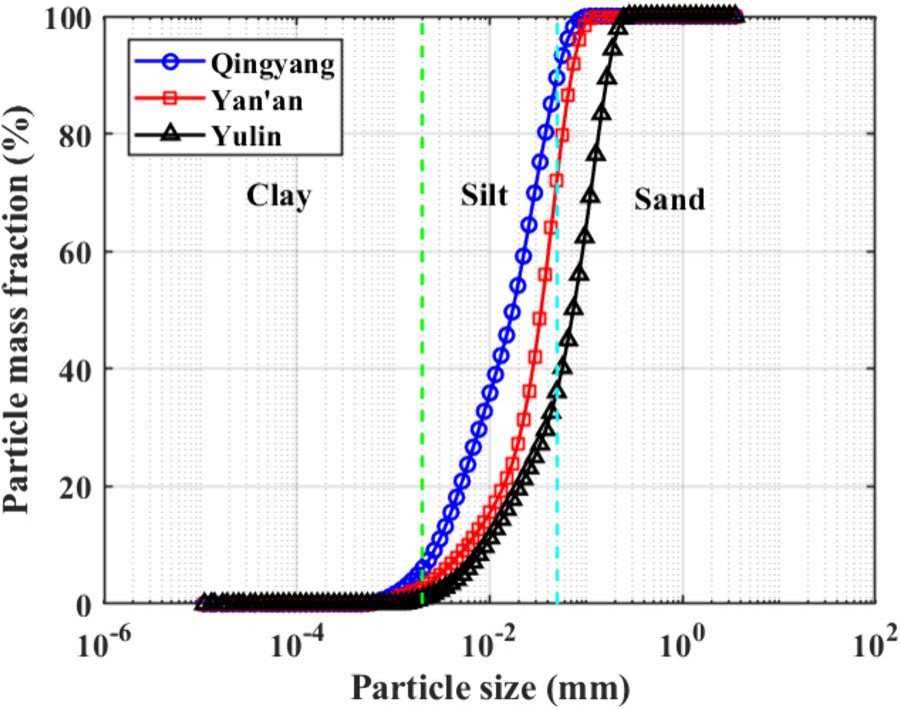
Particle distribution of soil sample composition.

The results of this particle-splitting experiment are shown in Fig 15.

Fig 15 shows that the clay content of the Qingyang soil samples was around 6.10%, that of the Yan’an soil samples was about 3.08%, and that of the Yulin soil samples was about 0.97%. Regarding overall composition, Qingyang soil samples had the highest percentage of tiny particles, followed by Yan’an and Yulin.

## Discussion

### Regional variability of loess

**Fig 16:**
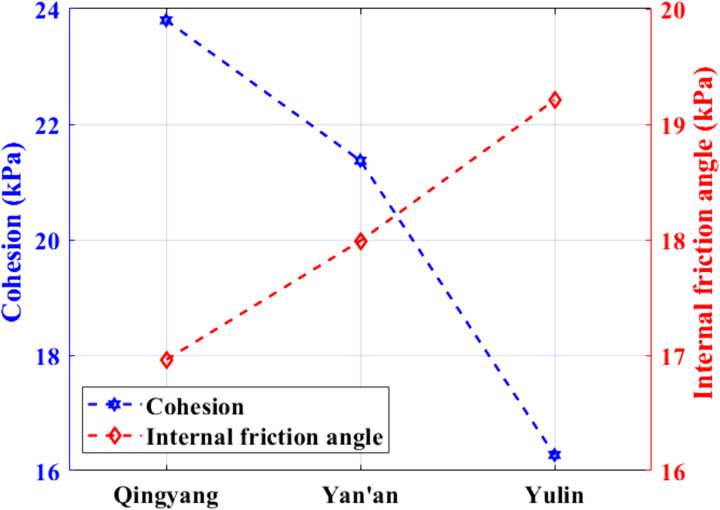
Loess strength index by region.

Fig 16 shows the statistics of the loess strength indexes in each region. As shown in Fig 16, the numerical magnitude of the cohesive force c is in the following order: Qingyang > Yan’an > Yulin; the numerical magnitude of the angle of internal friction Φ is in the following order: Qingyang > Yan’an > Yulin.

In general, there is an apparent correlation between soil strength and physical indexes such as water content and soil compactness. Still, the physical indices of the soil in the three test sites shown in Table 1 do not differ significantly, so the differences in the strength characteristics of the loess in the three areas should be more appropriately explained by the loess structure and combined with the changing rules of cohesion and internal friction angle of each soil sample shown in Figs 15 and 16, it is clear that there is a relationship between soil shear strength and soil particle composition. The internal friction angle decreases as the content of clay particles increases, whereas cohesion increases.

### Loess variability as influenced by clay particles

**Fig 17:**
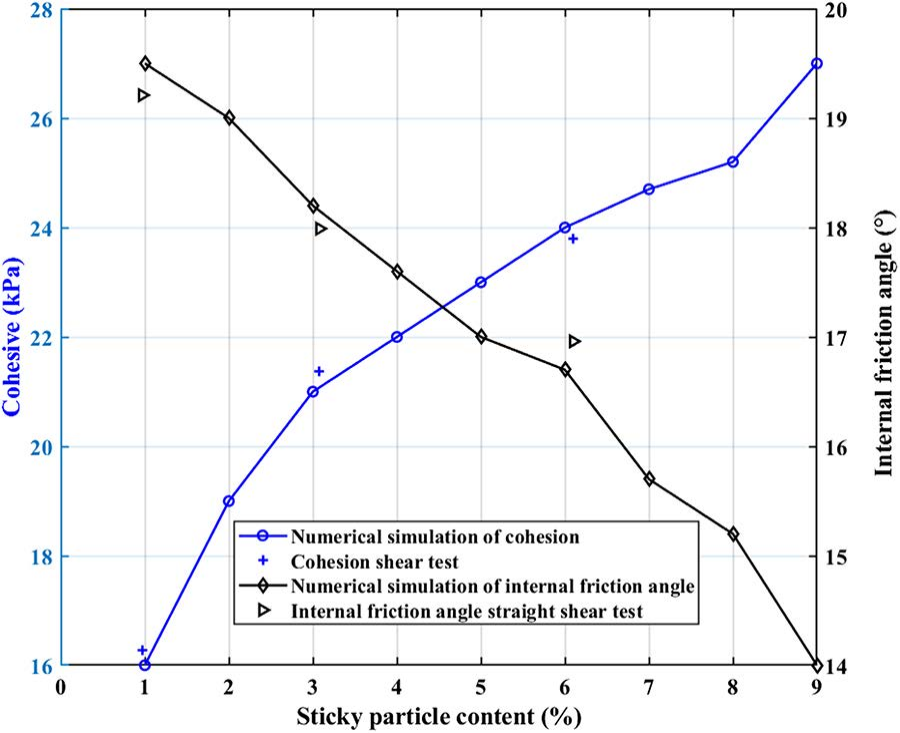
Relationship between strength indexes of loess and soil structure.

Through the discrete element model, the simulation results of the shear test under different particle gradations of loess, shown in Fig 17, can be obtained, consistent with the in situ straight shear test results. From the discrete element simulation results, the difference in loess structure in different regions is the primary intrinsic factor causing the change in the strength index of loess in the various areas. When the loess clay grain content increases from 1% to 9%, the cohesive force rises from 16 kPa to 27 kPa, an increase of 11 kPa; the angle of internal friction decreases from 19.5°to 14°, a decrease of 5.5°.

In other words, when the loess particle grading curve shown in Fig 15 is shifted to the left, the clay content of loess increases, the sand content decreases, the cohesive force increases relatively, and the angle of internal friction decreases relatively. Still, the rate of change of the cohesive force is significantly higher than the angle of internal friction, which indicates that the cohesive force is more sensitive to the change of the soil structure, such as the clay content of loess.

In the in-situ trials, the average increase in the compositional content of cohesive clay particles in Qingyang, Yan’an, and Yulin was approximately 3%. There is an average rise of 17.36% in cohesion and a drop of 4.39% in angle of internal friction for every 3% increase in cohesive clay particle content. According to the numerical calculations, every 3% increase in loess clay-grain content results in an average 25% increase in cohesiveness and a 10% decrease in internal friction angle.

The comparative analysis indicates a strong correlation between the shear strength of soil and the composition of its particles. The larger the soil particles, the more irregular their shape and rougher their surface, resulting in increased shear strength. Typically, larger particles exhibit a poorer degree of rounding and polishing, increasing the internal friction angle φ of loess as the proportion of coarse-grained components in its gradation rises. The particle size influences the soil’s moisture dynamics, and coarser surfaces with larger voids facilitate greater moisture permeability. The mechanism by which moisture affects the shear strength of soils is also partially coupled with the effect of the composition of the soil grains. Secondly, clay particles are the primary cementing material of loess. With small particle size, large specific surface area, and high surface energy, clay particles are closer to colloidal particles and have adsorption capacity, so the cohesion c value of loess increases with the increase of the composition of clay particles in its gradation. This partly explains the linear increase in loess cohesion with depth or deposition age.

### Analysis of the straight shear deformation damage process of loess

In the straight shear deformation damage process, loess particles contact each other through the particles to transfer force, and the contact force in the system is gradually interconnected to form a load transfer channel. This kind of contact force channel network is intertwined within the particle system, that is, the force chain network of the soil. As shown in Fig 21, the particle flow model can calculate the soil’s particle force chain network, reflecting the soil skeleton’s force characteristics from the microscopic level. The thickness of the force chain in Fig 22 represents the size of the contact force, and the direction of the force chain represents the direction of the contact force transfer.

**Fig 18:**
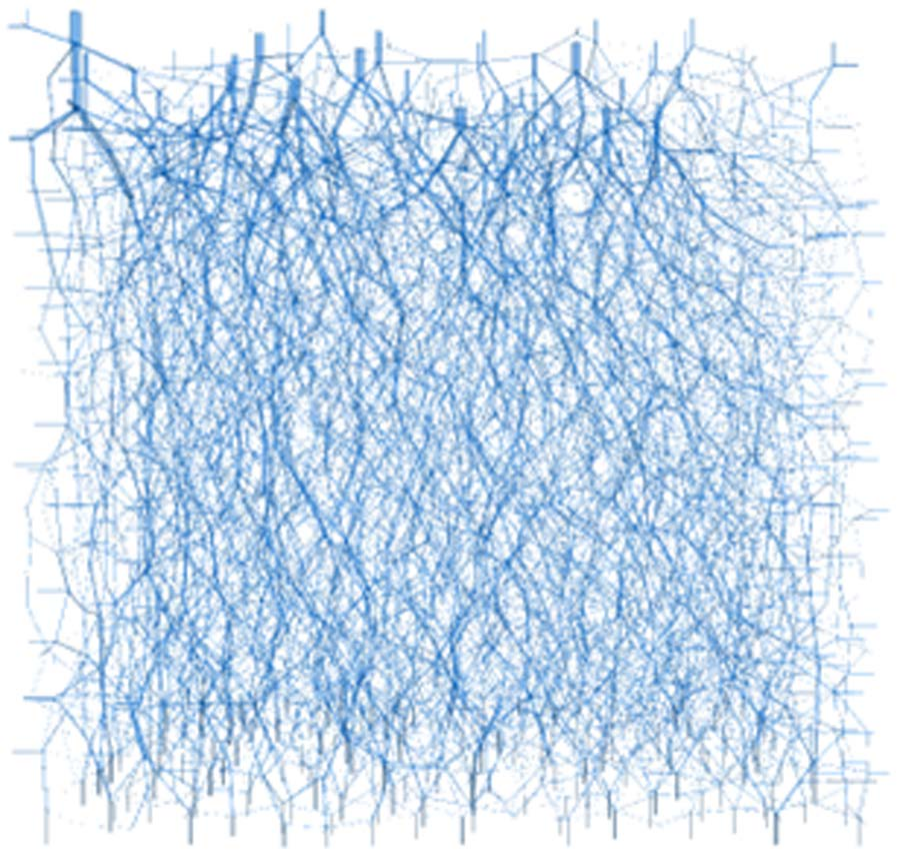
Force chain network Before shear loading.

**Fig 19:**
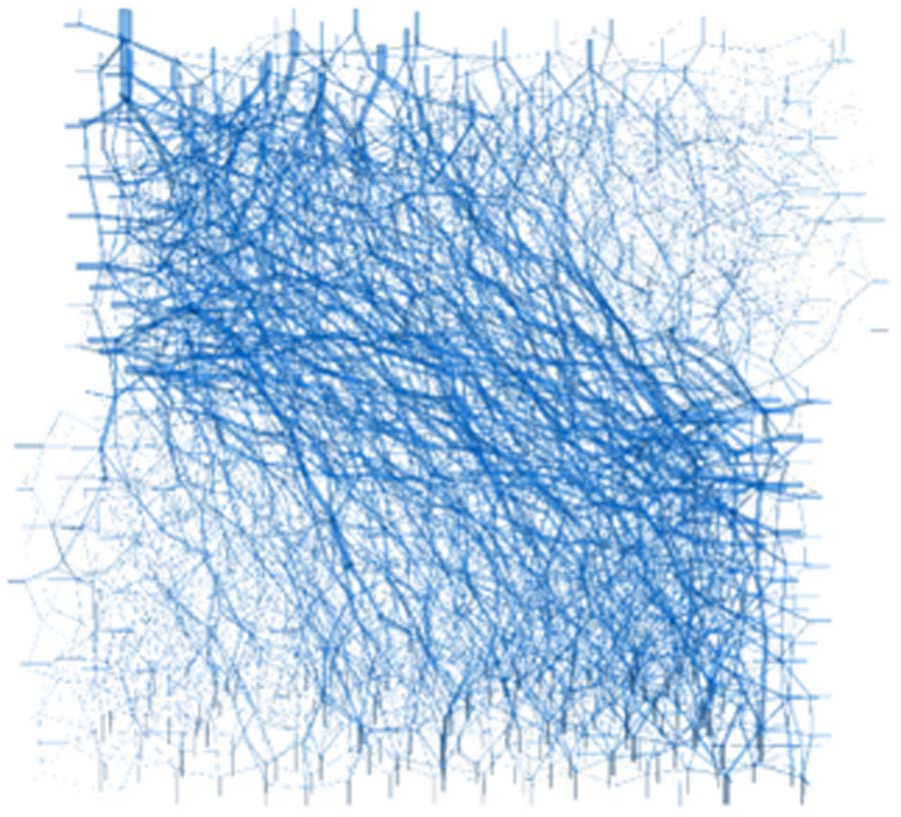
Force chain network After shear loading.

**Fig 20:**
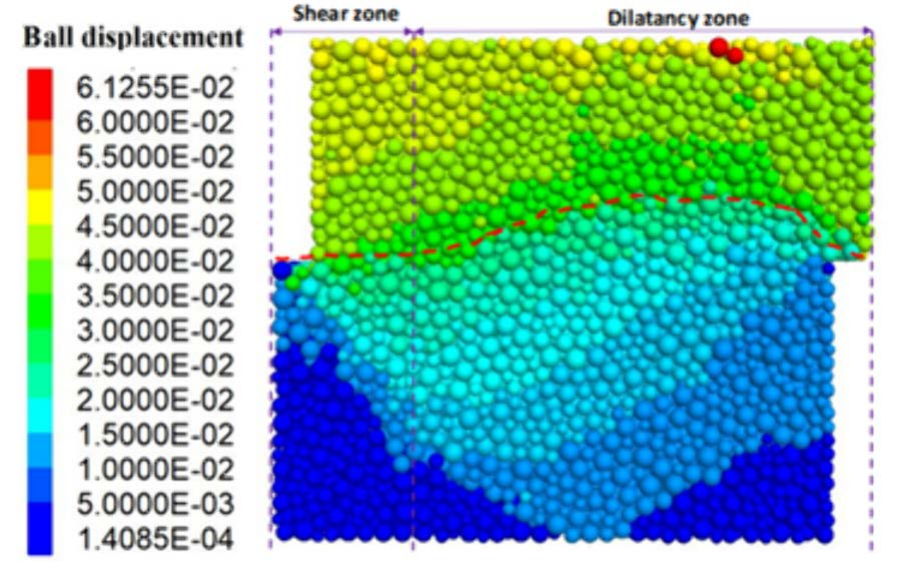
Displacement cloud map (geology).

**Fig 21:**
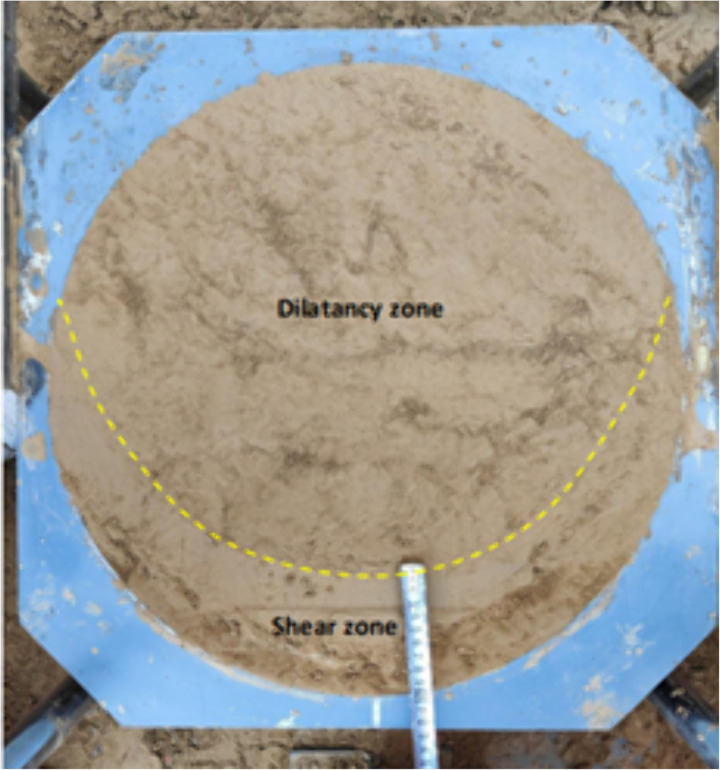
Shear-damage surface.

**Fig 22:**
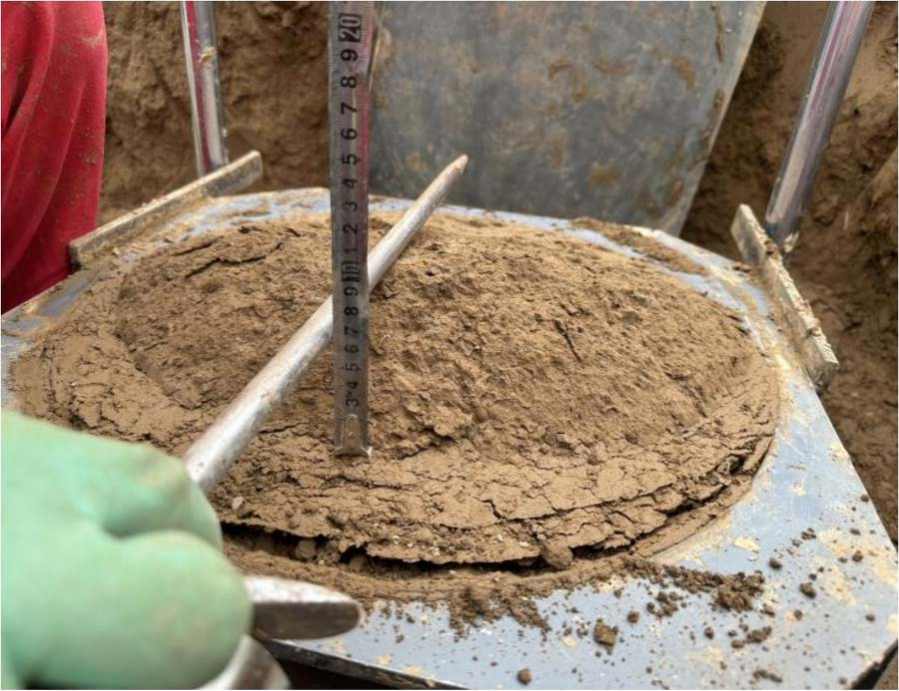
Bulging surface measurement process.

As shown in Figs 18 and 19, before shear, since the whole system is not subject to shear loading, only the contact force between particles exists inside the specimen, and the strong and weak force chains are interspersed and intertwined to form the whole force chain network. When the shear thrust is applied, the force chain network starts to be reassembled as the shear box is pushed, and the force chains gradually gather obliquely Fig 19. When the specimen hits the shear stress peak value, the force chain gathering reaches its maximum, resulting in the obvious obliquely inclined center chain belt, as well as the maximum angle of the central chain belt. Inside the loess specimen system, the contact between soil particles generates a force chain network that distributes and transfers external load, as well as the main force chain that adjusts the redistribution of the internal stress state and balances the influence of external loads. The process of force chain change shows that the force chain network is more sensitive to the change of shear loading and explains why the specimens in Figs 20 and 21 cannot form a completely horizontal shear damage surface.

According to the principle of statics, the misalignment between the upper and lower shear boxes will cause the soil sample to be damaged by shear and produce a horizontal shear damage surface between the upper and lower shear boxes. However, loess is a composite body with a special sedimentary structure, which is very different from the linear elastic rigid body assumed by statics. Fig 20 depicts specimen shear damage when the internal particle displacement cloud from the cloud is visible, with the loess specimen sandwiched between the top and lower shear boxes, resulting in a shear damage surface with a non-horizontal shape. However, a combination of surfaces with a transparent partition is approximately one-fifth the size of the shear horizontal surface and four-fifths the size of the push-shear bulging surface. As shown in Fig 22, the entire shear damage surface morphology is consistent with the in-situ test.

As demonstrated in the tests, the shear damage surface appears as a distinct bulging surface that has not been sheared horizontally. This can be attributed to the unique structural characteristics of loess: loess differs from the continuum material, is a kind of composite with specific particle gradation, maintains special depositional structural characteristics, and consists of solid particles, pore space, and water. The structural characteristics of loess lead to it in the straight shear test process, loess particles squeeze and slip each other, resulting in particles embedded in the pore space of the compaction process, as well as particles with each other “push”, “over” and “slip “Push and push”, “over” and “slip” between the particles to push and shear expansion process.

### Comparative analysis

Much literature has shown that the particle composition of soil has a direct effect on the macroscopic strength index of soil [14]. According to Zhuang, J [26], the adequate shear strength of cohesiveness of saturated loess with different clay concentrations varies. The experiment’s applicability and generalizability were validated as the effective internal friction angle varied little with clay content, with maximum and lowest variances of 1.02°. Changes in collection sites, sample preparation techniques, and the experiment’s indoor location all have the potential to cause interference.

Cohesion generally increases with higher clay particle content, while the angle of internal friction decreases. As clay particle content increases and sand particle content decreases, cohesion rises relatively, and the angle of internal friction decreases relatively. The larger the soil grains, the more intricate the arrangement of the particles, resulting in the coarse particles at the shear surface being elevated, misaligned, rotated, and extracted, accompanied by volumetric changes in the soil, reorientation of the particles, and the fracturing of the coarse particles themselves, thereby making it more challenging to surmount the interparticle friction. The value of the internal friction angle Φ rises with the augmentation of the coarse-grained content of its gradation. At the same time, the clay particles are the primary cementing materials of loess with adsorption ability, and the cohesion c value of loess increases with the increase of the clay particle component in its gradation due to the initiated chemo-combination build-up of van der Waals’ forces between the clay particles with the formation of contact points. All of the above are consistent with the results obtained in this paper.

## Conclusion

(1) Loess strength indexes vary significantly among regions, and its structural characteristics significantly influence the mechanical characteristics of loess.
(2) A correlation exists between soil shear strength and factors such as soil structure, moisture content, and initial density, which is further demonstrated by the intricate interaction between soil shear strength and the composition of soil particles.
(3) Loess cohesion is more sensitive to changes in soil structure, particularly with regard to clay content: for every 3% increase in loess clay-grain content, cohesiveness increases by an average of 17.36%. A leftward shift in the loess particle grading curve indicates an increase in clay content, a decrease in sand content, an enhancement in cohesion, and a decrease in the internal friction angle. By contrast, there is a 4.39% decrease in the internal friction angle. According to numerical simulations, the cohesiveness is improved by 25% and the internal friction angle is decreased by 10% for every 3% increase in loess clay-grain content.
(4) The varying mechanical properties of distinct particles, including clay, powder, and sand, manifested during soil deformation and failure, directly influence the strength index.
(5) The loess specimen system conveys and transmits external loads via a force chain network established by the interparticle interaction among soil particles, creating a primary force chain belt that regulates the redistribution of internal stress and mitigates the effects of external loads.
(6) The conclusion enhances and refines the theory for assessing the dynamic characteristics of loess regions, providing a comprehensive method to reduce the expenses associated with classifying various loess areas. Concurrently, we are deficient in micro-level analysis due to the experimental approach’s limitations. Future research will utilize approaches from microscopic analyses, including electron microscopy, with stronger indicators. We will analyze the mechanisms affecting loess strength indicators from local and macro viewpoints.
